# Thrombolysis After Thrombectomy? Revisiting Intra‐Arterial Therapy in the EVT Era

**DOI:** 10.1111/ene.70315

**Published:** 2025-07-26

**Authors:** Diana Aguiar de Sousa

**Affiliations:** ^1^ Department of Neurosciences, Stroke Center Lisbon Central University Hospital – ULS São José Lisbon Portugal; ^2^ Faculdade de Medicina Universidade de Lisboa Lisbon Portugal; ^3^ Gulbenkian Institute of Molecular Medicine Lisbon Portugal

Three decades after the NINDS trial established intravenous thrombolysis (IVT) as the cornerstone of acute ischemic stroke treatment [1], the field has made remarkable advances. Endovascular thrombectomy (EVT) has revolutionized care for large vessel occlusion (LVO) strokes. Evidence supports the continued use of IVT before EVT even in the mothership setting [2]. However, as EVT becomes more refined and widely implemented, new questions emerge: When successful recanalization has already been achieved, is there a role for adjunctive thrombolysis?

Intra‐arterial thrombolysis (IAT), once a primary method of reperfusion [3], fell out of favor with the rise of stent retrievers and aspiration techniques. Nonetheless, the observation that technically successful EVT does not always lead to favorable clinical outcomes has rekindled interest in IAT as a potential adjunct to improve the recanalization of distal vessels and microvascular perfusion.

In this systematic review and meta‐analysis, Palaiodimou et al. [4] assess the available evidence on the efficacy and safety of adjunctive IAT following successful EVT. Drawing on data from seven randomized controlled trials involving over 2000 patients, the authors report that IAT was associated with a higher likelihood of excellent functional outcomes at 90 days. A modest benefit was also seen in mRS score shift analysis, indicating reduced overall disability. Importantly, there were no significant differences in rates of symptomatic intracranial hemorrhage, mortality, or good functional outcome. The effect was consistent across subgroups, including patients who received IVT and those with complete or near‐complete angiographic reperfusion (eTICI 2c/3).

While the biological rationale is sound, as residual distal occlusions or impaired microvascular flow may persist after EVT [5, 6], several limitations must temper interpretation. First, the included trials have considerable variability in patient selection, thrombolytic agent, and dose. Although statistical heterogeneity was low, clinical heterogeneity was notable and may affect generalizability. Second, subgroup analyses revealed no clear dose–response effect or difference across reperfusion grades, raising questions about which patients derive the most benefit. Third, as the authors acknowledge, the analysis is based on aggregate‐level data, limiting the ability to explore individual‐level modifiers of treatment response.

Given the heterogeneity in study populations and treatment protocols, routine implementation of IAT after EVT cannot yet be recommended. Importantly, ongoing randomized trials, such as TECNO (NCT05499832), CHOICE‐2 (NCT05797792), and IA‐SUCCESS (NCT06768138), are expected to refine our understanding of the potential role of adjunctive IAT and how best to implement it. Until their results become available, clinical practice should prioritize enrollment in these studies rather than routine off‐label use of adjunctive IAT, as clinical equipoise remains and supports continued investigation.

While EVT has transformed stroke care, it has not rendered thrombolysis obsolete. This analysis offers a timely contribution to the ongoing discussion about whether adjunctive IAT can improve outcomes beyond what is achieved after mechanical thrombectomy alone. As we continue to refine our tools and deepen our understanding of cerebral reperfusion, a thoughtful reassessment of old strategies within the framework of modern stroke workflows is both appropriate and necessary. Defining the optimal combination and sequence of reperfusion strategies will be key to maximizing recovery for patients with ischemic stroke due to LVO.

## Author Contributions

**Diana Aguiar de Sousa:** writing – original draft, conceptualization.

## Conflicts of Interest

Dr. Diana Aguiar de Sousa reports research grants from FCT, MSD, and Astrazeneca Foundation, personal fees from Bayer, Daiichi‐Sankyo and Johnson & Johnson for advisory board participation, and speaking fees from Bial and Astrazeneca, outside the submitted work.

## Linked Articles

The added benefit of intra‐arterial thrombolysis after successful recanalization by endovascular treatment: A systematic review and meta‐analysis of randomized‐controlled clinical trials, https://doi.org/10.1111/ene.70270.

## Data Availability

Data sharing is not applicable to this article as no datasets were generated or analyzed during the current study.
